# SmartFluo: A Method and Affordable Adapter to Measure Chlorophyll *a* Fluorescence with Smartphones

**DOI:** 10.3390/s17040678

**Published:** 2017-03-25

**Authors:** Anna Friedrichs, Julia Anke Busch, Hendrik Jan van der Woerd, Oliver Zielinski

**Affiliations:** 1 Institute for Chemistry and Biology of the Marine Environment, Carl von Ossietzky University Oldenburg, Schleusenstrasse 1, 26382 Wilhelmshaven, Germany; jbusch@mpi-bremen.de (J.A.B.); oliver.zielinski@uol.de (O.Z.); 2 Life Sciences and Chemistry, Jacobs University, Campus Ring 1, 28759 Bremen, Germany; 3 Institute for Environmental Studies, VU University Amsterdam, De Boelelaan 1087, 1081 HV Amsterdam, The Netherlands; h.j.vander.woerd@vu.nl

**Keywords:** citizen science, affordable sensors, marine environment, open laboratory ware, LED, 3D printing

## Abstract

In order to increase the monitoring capabilities of inland and coastal waters, there is a need for new, affordable, sensitive and mobile instruments that could be operated semi-automatically in the field. This paper presents a prototype device to measure chlorophyll *a* fluorescence: the SmartFluo. The device is a combination of a smartphone offering an intuitive operation interface and an adapter implying a cuvette holder, as well as a suitable illumination source. SmartFluo is based on stimulated fluorescence of water constituents such as chlorophyll *a*. The red band of the digital smartphone camera is sensitive enough to detect quantitatively the characteristic red fluorescence emission. The adapter contains a light source, a strong light emitting diode and additional filters to enhance the signal-to-noise ratio and to suppress the impact of scattering. A novel algorithm utilizing the red band of the camera is provided. Laboratory experiments of the SmartFluo show a linear correlation (R${}^{2}$ = 0.98) to the chlorophyll *a* concentrations measured by reference instruments, such as a high-performance benchtop laboratory fluorometer (LS 55, PerkinElmer).

## 1. Introduction

Chlorophyll *a* fluorescence is well investigated and commonly used as a proxy for phytoplankton biomass [1] indicating the state of a marine water body, e.g., oligotrophic, mesotrophic or eutrophic [2]. Different processes are related to the state of algal biomass, such as net primary production [3] and harmful algal blooms [4]. An understanding of the marine algal biomass is a key element of the European Union marine monitoring framework and addressed by legislation such as the Marine Strategy Framework Directive (European Union 2008 [5]).

Stimulated chlorophyll *a* fluorescence measurements are widely applied [6,7,8,9]. The fluorescence emission process occurs randomly [10] and is defined as the ratio of moles photons emitted as fluorescence divided by the moles photons absorbed by all pigments [11]:

(1) $$\Phi =\frac{\mathrm{photons}\;\mathrm{emitted}}{\mathrm{photons}\;\mathrm{absorbed}}.$$

Quantum efficiency (Φ) of chlorophyll *a* fluorescence amounts to one to three percent [11,12,13] of the excitation intensity. Strength of the fluorescence signal is, on first approximation, dependent on the concentration of photo-active pigments in the sample and intensity of the excitation source (see Equation (1)). However, other mechanisms can impact the quantum efficiency Φ, such as quenching processes [14], photoinhibition [13], cell physiology [11], and photo-adaption processes e.g., photo acclimation and packaging effect [15]. Nevertheless, at least at moderate concentrations, a linear proportionality between chlorophyll *a* concentration and chlorophyll *a* fluorescence can be expected, but frequent calibration of this linear relation is required since the above described factors can alter the signal strength and linearity [16]. In addition, because algal pigments can absorb radiation near 685 nm [17] (self-shading effect), small deviations from linearity can occur at higher chlorophyll *a* concentrations due to absorption of the fluorescence signal.

The development and improvement of instruments based on the fluorescence phenomenon is ongoing and ranges from expensive laboratory-based to low-cost citizen instruments. In addition to high-resolution and high-end laboratory instruments, a large number of portable fluorometers for field experiments are commercially available [18,19]. These are used from various platforms [20,21] and a vast number of measurements are available in oceanology and limnology. One major drawback is, however, the high investment and maintenance costs for the use of fluorometers in large operational setups. However, especially in coastal areas, processes related to phytoplankton require highly resolved spatial-temporal measurements, as these waters tend to be highly dynamic and patchy in time and space [22].

Affordable portable fluorometers have the potential to significantly increase the number of users and measurements, thereby enhancing the coverage of coastal areas to detect phytoplankton related processes [23]. As yet, only a few affordable and portable sensors allow measurements of the marine realm by the general public. One promising example is presented by Leeuw et al. [24]: a low-cost (approx. 150 US $) in situ fluorometer. Each fluorometer, regardless of the type (fixed or portable), includes, but is not limited to, an excitation source and a detector. The detection of the fluorescence signal mainly depends on three parameters: (a) light intensity of the excitation source at relevant absorption wavelength; (b) quantum efficiency of the fluorescence process as an inherent optical property; and (c) sensitivity of the detector.

The usage of a smartphone, beyond its data logger capacities as an affordable and portable fluorescence sensor, is challenging. Its potential has been assessed in the European Commission 7th Framework Program (EU FP7) funded project CITCLOPS (Citizens’ Observatory for Coast and Ocean Optical Monitoring [25]) by answering the following questions: (Q1) How can the principle of measuring fluorescence be transferred to smartphones? (Q2) How can fluorescence be excited and detected by means of smartphone components? (Q3) Is the smartphone camera feasible as a detector for fluorescence? These questions will be addressed throughout the paper.

We present a proof-of-principle of an easy-to-use, affordable fluorescence sensor (SmartFluo) based on smartphone elements. It is designed as ‘do-it-yourself’ (DIY) sensor system (approximately 50 EUR excluding smartphone) to encourage citizens to perform environmental observations with their smartphone. This includes construction of the sensor with easy-to-obtain and affordable components, an integration of internal smartphone elements wherever possible, and a robust algorithm to convert the fluorescence signal to chlorophyll *a* concentrations. This proof-of-principle of measuring fluorescence by means of smartphones is an important step towards inclusion of smartphones into field data recording.

## 2. Method

This section explains three different parts of the newly developed method, which includes the hardware (Section 2.1), the novel algorithm (Section 2.2) and finally the laboratory validation (Section 2.3).

### 2.1. Hardware

**Figure 1:**
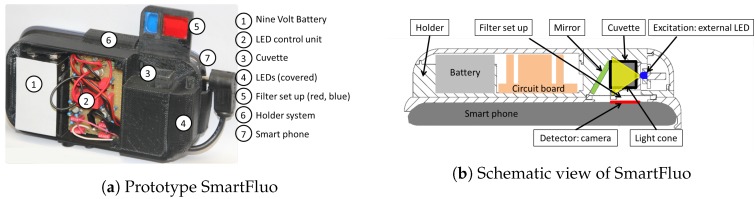
Open view (**a**) and schematic drawing (**b**) of the SmartFluo prototype.

*(Q1) How can the principle of measuring fluorescence be transferred to smartphones?* Five key elements concerning hardware and software were defined: most crucial are (1) excitation source and (2) detector, in addition for equality of the measurement (3) a sample holder and (4) an ambient light protection, and above all (5) a fluorescence signal compiler. For compilation, an algorithm (see Section 2.2) was developed to analyse detected signals. To accommodate the key elements one to four, all components were concatenated into a small measuring add-on to the smartphone: SmartFluo (see Figure 1a). It was designed for the smartphone Samsung Galaxy S3 mini (Samsung, Seoul, Korea). This widely distributed smartphone and operating system (Android version 4.1.2, Google, Mountain View, CA, USA) allows a transferability of the method to other devices.

In accordance with open source protocols, an affordable add-on was constructed with computer-aided drafting, prepared with SolidWorks (Dassault Systèmes SolidWorks Corp., Waltham, MA, USA), and printed on a three-dimensional (3D) printer (MakerBot^®^ Industries LLC, New York, NY, USA). Within the add-on, excitation source and detector were typically aligned in a 90-degree setup (see Figure 1b) to minimize scattering effects on the detected signal [10,13].

### 2.2. Algorithm

A simple algorithm (RGB2Chl) was developed for extraction of the chlorophyll *a* fluorescence signal from red-green-blue (RGB) images of the camera and conversion to chlorophyll *a* concentration. This RGB2Chl algorithm is based on three assumptions (1) the photons of fluorescence emission can be captured by the red channel of the RGB image (digital number of red channel); (2) the photons absorbed are related to the illumination time of the image; and (3) the fraction of emitted light to absorbed light is constant. Then, the chlorophyll *a* concentration is proportional to the intensity of the red channel ($I_{red}$), determined by:

(2) $$I_{red}=\frac{\mathrm{digital}\;\mathrm{number}\;\mathrm{of}\;\mathrm{red}\;\mathrm{channel}}{\mathrm{illumination}\;\mathrm{time}}.$$

The S3 mini smartphone camera takes RGB images with a resolution of 2560 × 1920 pixels in JPEG (Joint Photographic Experts Group) format with a respective illumination time determined by internal image optimization algorithms. Internal S3 mini camera settings convert automatically the irradiance (photons m${}^{-2}$·nm${}^{-1}$) into digital numbers (DN) [26] leading to a new unit DN·s${}^{-1}$ for $I_{red}$. Conversion process from raw-images of the camera to RGB images takes place as an internal process [26] following the colorimetric standards worked out by Commission Internationale de l’Eclairage 1931 XYZ color space [27,28,29].

The exact relationship between $I_{red}$ and the chlorophyll *a* concentration depends on the quantum efficiency. To obtain $I_{red}$, the RGB2Chl algorithm coded in Matlab 2012b (The MathWorks^®^, Inc., Natick, MA, USA) was applied to RGB images in the following steps:

1. **Selection of sub image** (region of interest): As the geometry of the measurement and the focus field of the camera is constant, a 300 × 300 pixel field at the centre of the image is selected to avoid catching edge effects of the cuvette. The detected signal proved to be stable over this region.
2. **Red channel extraction**: The digital number of the red channel is determined for each 300 × 300 pixel field of the RGB images.
3. **Illumination time extraction**: The illumination time is read out from the property information of each image and saved for further analyses.
4. **Intensity calculation**: For each image, $I_{red}$ is calculated after Equation (2).
5. **Blank correction**: Intensity of the sample is corrected by subtracting the intensity of a blank reference sample (base-line correction).

### 2.3. Laboratory Validation

*(Q2) How can fluorescence be excited and detected by means of smartphone components?* The characteristics of the SmartFluo were compared with commercially available instruments for excitation and detection of chlorophyll *a* fluorescence. Performance of the excitation source was described by the emission intensity and wavelength range. The detector capacity was tested for its ability to detect red light at low intensity with high signal-to-noise ratio. The performance of the SmartFluo prototype and algorithm were tested with a dilution series of chlorophyll *a* standard (5000 μg·L${}^{-1}$ dissolved in 90% acetone) under laboratory in vitro conditions for demonstration and controlled assessment reasons. Dilution series covered a range of 1–250 μg·L${}^{-1}$ in intervals of 1 (1–10 μg·L${}^{-1}$), 5 (10–50 μg·L${}^{-1}$) and 50 (50–250 μg·L${}^{-1}$) μg·L${}^{-1}$ steps measured in a quartz glass cuvette of 1 cm path length (Hellma Analytics, Mühlheim an der Ruhr, Germany). With the SmartFluo, every dilution step was measured in triplicate to determine stability of the method. Pure 90% acetone was used as blank for baseline correction [30]. Spectral intensity and signature of the excitation source were measured by a hyperspectral radiance sensor (Ramses-ARC-vis, TriOS, Rastede, Germany), while intensity was compared to the commercial instrument, MicroFlu Chl-A (TriOS), both calibrated to the manufacturer’s calibration spectrum based on National Institute of Standards and Technology (NIST) standards. After the algorithm was applied, the intensity of fluorescence $I_{red}$ was calibrated to the pre-determined chlorophyll *a* concentration.

*(Q3) Is the smartphone camera feasible as detector for fluorescence?* The performance of the smartphone camera was evaluated by laboratory experiments with the above described dilution series. Simultaneous measurements with a benchtop fluorescence spectrometer equipped with a red-sensitive R928 photomultiplier (LS55; PerkinElmer Ltd., Waltham, MA, USA) served as proof of concept and control measurements. Instrument settings (see Table 1) were selected in order to measure all dilution steps of the chlorophyll *a* standard in a similar fashion. Performance of the smartphone camera as fluorescence detector and the RGB2Chl was compared with that of the LS55. According to Arar and Collins [31], a linear regression was performed for quality control.

Laboratory experiments for excitation source, detector and SmartFluo were conducted and are presented in Section 3.1, Section 3.2 and Section 3.3, respectively. All the results regarding chlorophyll *a* data were performed under in vitro conditions.

## 3. Results

### 3.1. Excitation and Detection

For the selective method of measuring the concentration of chlorophyll *a* by means of fluorescence, excitation source and detector are the most crucial elements. Chlorophyll *a* fluorescence is commonly induced by blue light with peak wavelengths near 440 nm for in vivo or 430 nm for in vitro measurements the primary absorption maxima of chlorophyll *a*. The resulting fluorescence signal is emitted in visible red light range with peak wavelength of approx. 685 nm and 668 nm for in vivo and in vitro measurements, respectively [13,17], with Gaussian distribution of 25 nm bandwidth around the centre wavelength [11].

(Q2) How can fluorescence be excited and detected by means of smartphone components?

**Figure 2:**
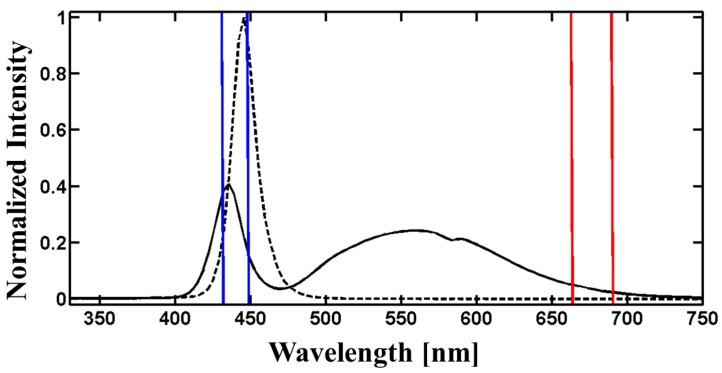
Spectra of internal smartphone LED (black continuous line) and external blue LED (black dashed line) including interesting spectral blue and red wavelength ranges (lined with blue and red lines, respectively).

**Figure 3:**
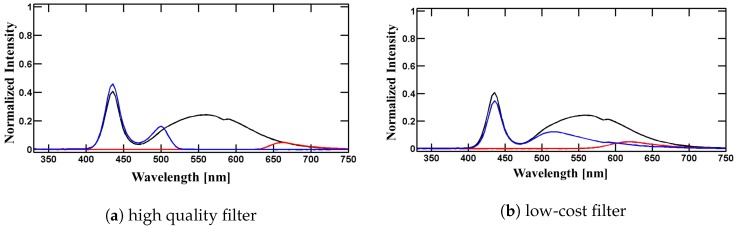
Normalized spectra from the internal smartphone LED after transmission through (**a**) high-quality filters and (**b**) low-cost filters. These spectra were measured to test the amount of light available for excitation after restriction with a blue filter (blue lines) and how much light is still reaching the detector protected by a red filter (red lines).

*Excitation*: Two alternative light sources were examined as excitation sources for chlorophyll *a* fluorescence: (a) the internal S3 mini smartphone light emitting diode (LED) (flashlight) and (b) an external blue LED (see Figure 2, dashed line). The spectral signature of the S3 mini smartphone LED is characterized by two significant peaks at 439 nm around 570 nm (see Figure 2, continuous curve). Sharpening of the spectral signature was successfully accomplished by application of filters restricting excitation light to the desired blue fraction (430 nm to 450 nm, see Figure 3a,b). Efficiency of a blue low-cost transparency filter (#69, Rosco, Stamford, CT, USA) was compared against a high-quality filter (type IF460, bk Interferenzoptik Elektronik, Nabburg, Germany) as used in commercial instruments. However, the intensity of S3 mini LED combined with low-cost filter (74 mW·m${}^{-2}$·nm${}^{-1}$·Sr${}^{-1}$) or with high-quality filter (92 mW·m${}^{-2}$·nm${}^{-1}$·Sr${}^{-1}$) was not comparable to the intensity of the commercial instrument operating higher than 550 mW·m${}^{-2}$·nm${}^{-1}$·Sr${}^{-1}$. Therefore, an external affordable blue LED (LUXEON Rebel LED, Philips Lumileds Lighting Company, Amsterdam, The Netherlands) with a sufficiently high intensity (approx. 580 mW·m${}^{-2}$·nm${}^{-1}$·Sr${}^{-1}$) at the peak wavelength of 448 nm (see Figure 2, dashed line) was chosen. Despite the elegance of using the internal S3 mini smartphone LED as a light source, the usage of the smartphone LED had to be discarded and SmartFluo was designed with an external LED. This provided the advantage that no external filter was required for excitation.

**Figure 4:**
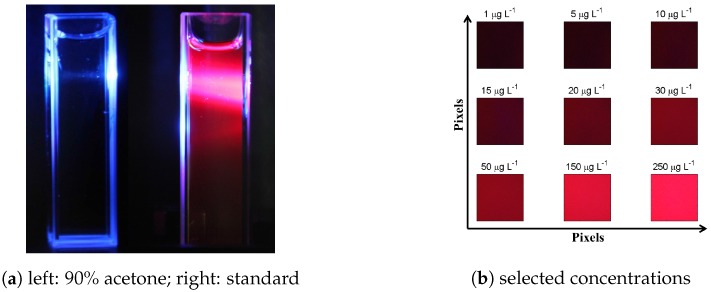
Red-Green-Blue images taken by a commercial digital single reflex camera of (**a**) clear 90% acetone (**left**) and chlorophyll *a* standard (**right**) illuminated by an external blue LED and (**b**) RGB images (300 × 300 pixels) of fluorescence signal of selected chlorophyll *a* concentrations obtained by the SmartFluo.

*Detection*: We aimed for the use of the internal smartphone camera as detector. Preliminary tests with a digital single reflex camera (Canon, EOS 60D, Tokyo, Japan) detector indicated that changing red fluorescence intensity with changing chlorophyll *a* concentrations can be reproduced on RGB images—for instance, an RGB image of pure acetone compared to chlorophyll *a* standard (see Figure 4a). Tests were successfully reproduced with the Samsung Galaxy S3 mini smartphone camera (see Figure 4b), which was therefore chosen as the detector in the SmartFluo. Despite weak visible differences in reddish tones, especially in the case of lower chlorophyll *a* concentration, the algorithm detects differences. Due to limited camera setting possibilities, scene mode was set to ‘automatic’ and white balance to ‘daylight’. To avoid corruption of the rather weak fluorescence signal from ambient light, all non-fluorescence signals or scattered light of the excitation source, a red filter was used in front of the camera. An efficiency test of a red, low-cost transparency filter (#19, Rosco) was conducted in comparison to a high-quality filter (type RG645, bk Interferenzoptik Elektronik, Nabburg, Germany) used in commercial instruments. Since the external LED has no red fraction (see Figure 2, dashed line), both filters suppressed the transmission of external blue LED light under 0.1% (data not shown). Therefore, the red low-cost transparency filter was chosen as suitable of being integrated into the three-dimensional printed holder.

### 3.2. Affordable Adapter

All elements of the add-on were selected to be easy-to-obtain and affordable (see Table 2). Integration of external LED elements required additional mechanical and electro-optical components within the add-on to control the external LED. A small voltage-circuit was designed to give a stable voltage of approximately 3 V and to switch the external LED on and off by means of a manual switch. Alternatively, the excitation source could be triggered by an application program (APP). The trigger-signal is sent through the earphone plug-in to the external LED (see Figure 1a) and receives a constant power supply based on an integrated circuit.

### 3.3. Laboratory Experiments—Proof of Concept

*(Q3) Is the smartphone camera feasible as a detector for fluorescence?* An essential feature of SmartFluo prototype performance is the capability of the smartphone camera. This is analysed by the performance of the SmartFluo and the related newly developed RGB2Chl algorithm, and proved by proof of concept.

**Figure 5:**
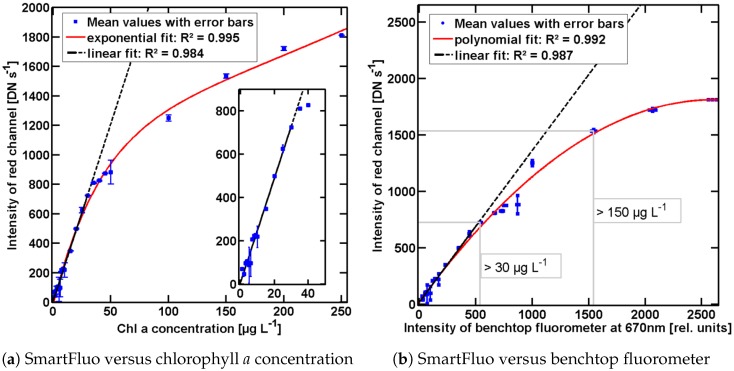
Intensity of the red channel of the smartphone as *a* function of (**a**) chlorophyll *a* concentration and (**b**) maximum intensity of the LS55 at 670 nm. Triplicates (blue dots) are shown including error bars (blue squares). In both cases, a linear fit [black, R^2^ = 0.98 and R^2^ = 0.99] is applied for low chlorophyll *a* concentrations: 1 μg·L^−1^ to 30 μg·L^−1^, highlighted in (**a**). Dashed parts of fitting line are not included into the fit. The red curve in (**a**) describes exponential fit (R^2^ = 0.98), and in (**b**) a polynomial fit (R^2^ = 0.99) over the full dilution series. In (**b**), related chlorophyll *a* concentrations are additionally marked in two ranges: >30 μg·L^−1^ and >150 μg·L^−1^ (grey boxes).

*Performance of SmartFluo and RGB2Chl*: The calculated $I_{red}$ ascends (from 45 DN·s${}^{-1}$ to 1814 DN·s${}^{-1}$) with increasing chlorophyll *a* concentration, reaching a saturation at higher chlorophyll *a* concentrations (see Figure 5a). The SmartFluo instrument delivered a rather stable fluorescence signal (small deviations in triplicate) over a large range of chlorophyll *a* concentrations. Linear regression was fitted to a range of assumed linear relation and behaviour from 1 μg·L${}^{-1}$ to 30 μg·L${}^{-1}$ (embedded in Figure 5a, black line). A double exponential fit (coefficients: a = 1170, b = 0.001823, c = –1190, d = –0.04299), over the whole range (Figure 5a, red line) describes best the linear relation at low concentrations and the saturation process at higher chlorophyll *a* concentrations. Both fits provide a coefficient of determination (R${}^{2}$) higher than 0.98.

*Proof of concept*: Control measurements confirmed the applicability of the SmartFluo and the RGB2Chl as a new principle for measuring chlorophyll *a* fluorescence. A linear regression between intensity of in vitro fluorescence maximum of LS55 at 670 nm and $I_{red}$ (see Figure 5b, black line) was applied in expected linear ranges at low chlorophyll *a* concentrations (1 μg·L${}^{-1}$ to 30 μg·L${}^{-1}$). R${}^{2}$ of 0.99 confirmed the assumption that $I_{red}$ is properly recorded and determined correlating to the assumed linear behaviour of $I_{red}$. An additional polynomial fit (coefficients: a = –0.000263, b = 1.374, c = 18,7) represents the sensitivity curve of SmartFluo and RGB2Chl with R${}^{2}$ of 0.99 (see Figure 5b, red line).

SmartFluo shows a lower detection limit of 10 μg·L${}^{-1}$ based on evaluated linear regression. Precision (4.5 DN·s${}^{-1}$) is given on the basis of averaged standard deviation. The upper detection limit is fixed due to the kind of saturation effect of $I_{red}$ in higher chlorophyll *a* concentration (see Figure 5a), the dilution series ends at 250 μg·L${}^{-1}$. The method works best in the linear $I_{red}$ range up to 50 μg·L${}^{-1}$. Beyond the lower detection limit, a small standard deviation (1 DN·s${}^{-1}$ to 80 DN·s${}^{-1}$) promises a stable running system to measure chlorophyll *a* fluorescence by means of smartphones.

## 4. Discussion

SmartFluo compiles internal smartphone- and additional elements into a new sensor system for measuring chlorophyll *a* fluorescence by means of smartphones. It is designed to be used by citizens in coastal areas and therefore is built with low-cost components ensuring minimal reduction of precision. Technically, the excitation source and detector are carefully selected and tested. An external filter at the excitation side became redundant by use of a selected high-output blue external LED, confirming the potential of LED technology. However, this resulted in additional cost for a voltage-circuit. The selection of the external LED (peak wavelength at 448 nm) is based on a reasonable compromise of affordable available LEDs and best suitable excitation range for chlorophyll *a* fluorescence. The fact that many commercial instruments use LEDs that emit at longer wavelengths than the optimum at absorption maximum of in vivo chlorophyll *a* (at 440 nm [17]) can be traced back to the market availability of LEDs when production of these instruments had started. Nevertheless, the choice of wavelength affects mainly the quantum efficiency. The closer to the absorption maximum of chlorophyll *a* pigments, the higher is the expected fluorescence emission.

In addition to the excitation wavelength, over-illumination also affects quantum efficiency. At illumination times longer than a second, the photosystem becomes saturated, thereby reducing the efficiency. In SmartFluo, the illumination time is set below one second and as short as possible for triggering the system. Measurements are influenced by accuracy of the charge-couple device (CCD) chip of the camera and its settings e.g., scene mode and white balance. Therefore, usage of the same mode in all measurements is important. In particular, the white balance set as daylight mode is most relevant to reach the same RGB colouring for comparison, a well-defined white point [32,33], and to reduce errors [34]. Effects of megapixel count and quality of lenses which usually have an effect on image quality are not expected to influence $I_{red}$ as it is averaged over a fixed square of pixels independent of the megapixel count.

The use of the earphone plug-in port to connect the housing to the phone was selected due to its backward-compatibility, simplicity, and ubiquity [35], giving SmartFluo a broad range of applications in different types of mobile phones. Nonetheless, the smartphone type plays a role, since different smartphone types have different cameras and software control, thereby introducing a variation in quality of the measured fluorescence signal. Therefore, setting up fixed parameters within the APP is highly essential for the use of the algorithm in different smartphone types in order to avoid significant adaptions. When a different type of smartphone is used, specifically with a different camera, it is highly recommended to perform a quality control against a commercial fluorometer to check for performance of the SmartFluo. Regarding precision of the data, the measured triplicates gave information about stability of the SmartFluo and were used for error calculation and can be used as first quality check for functionality of the instrument. The additional control measurements also support the low bias and overall good performance of the SmartFluo. Good quality data can be taken by citizens for estimation of chlorophyll *a* concentration, especially at concentrations above 10 μg·L${}^{-1}$.

Results proved SmartFluo to be a valuable tool for measurement of extracted chlorophyll *a*. The affordable instrument has the potential to enhance the coverage of algal biomass measurements over time and space. The presented work is a major step towards the use of SmartFluo by scientists and citizens in field observations.

*Environmental influences*—Major aspects which need to be taken into account for future field application are disturbances of the fluorescence signal by light scattering by algal cells and other particulate material in water, and influences of absorption. In particular, coloured dissolved organic matter is highly absorbing. Due to the availability of light to be absorbed by algae changes, consequently, chlorophyll *a* fluorescence signal depends on the absence or presence of other absorbing or scattering particles. Additionally, the phytoplankton community composition has an impact on the fluorescence [36]. Therefore, with respect to field conditions, a detailed investigation is required for the relationship between chlorophyll *a* and $I_{red}$. It is well known that fluorescence quantum efficiency is somewhat dependent on temperature [13]. It is therefore recommended to keep the time between sampling and measurement as short as possible. The quantum efficiency is also affected by field conditions and cells’ appearance since absorption varies with them [36], but changes are in a reasonable range. Despite environmental influences, the SmartFluo is designed in a way that it enables reliable field measurements and estimations of chlorophyll *a* concentrations.

*Potential field applicability*—Regarding standard measurement methods of chlorophyll *a* , some aspects of field applications need to be considered. Sample preparation should be minimized. Filtering and dealing with acetone should be avoided to keep simplicity for users; therefore, SmartFluo is designed as an in situ fluorometer and its excitation intensity is comparable to commercial in situ fluorometers e.g., MicroFlu Chl-A (TriOS) used in LOW mode, assuming a high chlorophyll *a* concentration or other commercial fluorometers [37]. For field use of SmartFluo, three points need to be considered: (1) calibration of the instrument, for instance, with solid standard; (2) citizens without prior experience in water quality monitoring should get feedback about the data they measured; and (3) concerning the detection limits. The latter two points could be addressed by setting different ranges of chlorophyll *a* concentration instead of precise numbers to classify water samples as indicators for water quality. These aspects need to be considered in field investigations. A first quality check is performed by measuring triplicates, thereby supplementing other water quality monitoring parameters given by citizen tools such as ‘Eye on Water Colour’ or water transparency.

*Citizens’ science aspects*—Not only scientists, but also citizens should be able to prepare the instrument and perform measurements. SmartFluo was designed with easy available and affordable elements, following the trend of DIY instruments and experiments. A detailed instruction is openly available as supplementary information, and allows the construction or adaption of the instrument by any interested user. SmartFluo is hence open to the general public. Due to the increased effort in acquisition of construction elements, construction and measurement, addressed user groups are environmentally engaged groups and water quality monitoring institutes [38]. By use of 3D printing techniques and the small voltage-circuit, the range of possible user groups is extended from environmentally engaged to technical affine users, maker- and open lab-ware movements [38]. The use of smartphones as a tool for recording hydrological observations proved to be a success in the ‘Social.Water’ study [39]. The ‘Eye on Water Colour’ application [40] developed in project CITCLOPS shows that the colour of marine waters can be measured by citizens in an operational fashion. This application (APP) encourages citizens to participate in marine scientific measurements and to enhance their awareness of the marine environment [38].

Comparable to the ‘Eye on Water Colour’ APP, SmartFluo measurements are standardised by the use of 3D printed housing, a fixed excitation wavelength, an APP for well-defined camera settings e.g., illumination time, white-point, scene mode and evaluation algorithm. Hence, the possibility of human errors with SmartFluo are minimised.

The field of citizen science is enhanced by SmartFluo and supplements the ‘Eye on Water Colour’, the DIY fluorometer by Leeuw et al. [24] and other affordable sensors [38]. The DIY fluorometer [24] offers the possibility to integrate citizens but without smartphones and is noticeably larger than the handy smartphone with add-on (length × width × height: 13.5 cm × 4 cm × 7 cm). The mobility and stowage of the SmartFluo is an advantage over the DIY fluorometer by Leeuw et al. In addition, the use of smartphones and APPs in citizen science allows an automated creation and upload of standardised metadata along with each measurement [38]. Gathering the data collected by users is an essential part. Therefore, an uploading system as used for ‘Eye on Water Colour’ will be implemented into a final APP version of SmartFluo. The inclusion of citizens to environmental observations has recently gained recognition [41,42]. As early as 2010, Kuo et al. [35] already indicated mobile phones as the most robust basis for sensor systems. This is particularly attractive for citizens, especially if their personal smartphone can be converted into a sensor system.

SmartFluo is designed to be easily adoptable. Small changes in 3D printing design allows its use with different smartphone types. By exchanging the external LED, different substances or algal types could be excited: ultra-violet LED or a certain peak wavelength of a blue LED could be used to excite coloured dissolved organic matter [42] or cyanobacteria, respectively.

*Improvements and Recommendations*—All components of SmartFluo were carefully selected for field and citizen application. To account for changing environmental conditions in coastal regions depending on their surrounding area, field tests in different regions are required. Influences and effects in those regions are diverse; therefore, the method is developed to allow adjustments considering not only smartphone components but also changing environmental conditions e.g., investigation of coloured dissolved organic matter. The use of an APP instead of a manual switch will increase the standardisation and hence also the quality of chlorophyll *a* measurements by means of smartphones. Another improvement would be the implementation of RGB2Chl into such an APP. Especially displaying the data right after the measurement on the screen could enhance the motivation and hence mobilization of citizens. Furthermore, variations in the spectral range of excitation light source would allow for specification of different algal groups [43]. In the beginning of the ‘smartphone sensor system era,’ it is recommended to use commercial instruments and reference measurements for comparison and further improvements, if available. Nevertheless, SmartFluo opens up opportunities for further applications in the field of bio-optical oceanography.

## 5. Conclusions

In this paper, the SmartFluo prototype and the RGB2Chl algorithm that together constitute an easy to handle, portable and affordable method for fluorescence measurements by smartphones is presented. The conversion of a smartphone into a fluorescence sensor system broadens the scientific user groups and inspires citizens to perform environmental measurements. Usage of internal smartphone elements is a particular strength of the method. One novel aspect is the use of the internal smartphone camera as a detector. Due to the flexibility of the three-dimensional print technology, the external holder of batteries and blue LED can be adapted to other smartphone types. For more information, see the additional material. While the use of the add-on is straightforward and the single components for building are easy to obtain and in an affordable price range (see Table 2), it requires some degree of technical skills and electronics to construct the add-on from all components.

Its easy handling offers citizens a possibility to conduct chlorophyll *a* measurements with their smartphones that can feed directly into existing chlorophyll *a* fluorescence data sets. In addition, internal smartphone sensors allow an automated detection of relevant metadata, such as position and time of each measurement. These can be added to the measurement output in a separate metadata format. In CITCLOPS, these are already designed and stored in the required format for an automated upload to international data repositories, such as EMODnet (European Marine Observation and Data Network) or SeaDataNet [38].

Thus, SmartFluo opens possibilities to address research questions, especially in coastal areas, by using data collected by citizens. This allows identification of new regions of interest for further investigations e.g., rapid changes in biomass in coastal areas based on quick estimations of chlorophyll *a* fluorescence. In this way, more precise, time-consuming and high-end fluorescence measurements could be applied efficiently and in problem areas, based on SmartFluo applications. It also enables a quasi-synoptical and large spatio-temporal data set.

SmartFluo is designed as an instrument to be used by citizens to estimate the chlorophyll *a* content in natural waters, but cannot reach the sensitivity and precision of high-end laboratory equipment. Accuracy is also limited by hardware components and excitation intensity. Therefore, SmartFluo does not replace but rather supplements high-end instruments. In conclusion, we find that the new concept of using smartphones as sensor systems works well for measuring chlorophyll *a* fluorescence. It can be detected by the smartphone camera, and the algorithm delivers a promising estimation of the chlorophyll *a* concentration with surprisingly high precision at higher concentrations.

## Figures and Tables

**Table 1:** Instrument settings for methods used for validation of SmartFluo. $\lambda_{Ex}$ and $\lambda_{Em}$ represent excitation and emission wavelengths, respectively.

Parameter	Instrument	Settings	Purpose
Fluorescence	Adapter	White balance: daylight; Focus: automatic; Illumination time <1 s	Method to be validated
Fluorescence	LS55	$\lambda_{Ex}$: 448 nm; $\lambda_{Em}$: 600–750 nm	
		Gain 775 V to 950 V	Control measurements
		Scan Speed: 1000 nm·min${}^{-1}$	
		Emission and Excitation slit: 10 nm	

**Table 2:** List of components required in addition to the smartphone. Components of the voltage-circuit are condensed (detailed information given in Supplementary File).

Component	Manufacturer	Price [Euro]
3D printed holder	not specified	10–50
External LED	Philips Lumileds Lighting Company, Amsterdam, The Netherlands	5.00
Voltage-circuit	various	16.63
Cuvette	Brand, Wertheim, Germany	0.11
Transparency filters	Rosco, CT, USA	10.97
**Total costs approximately**		33.00–73.00

## Supplementary Materials

The following are available online at www.mdpi.com/1424-8220/17/4/678/s1, Figure S1: Circuit diagram of the LED control board including suggested connection points for battery-clip cable. Circuit set up is for a 100 mA LED-current (IC1). Produced with EAGLE CadSoft by Rohan Henkel, Figure S2: Circuit board with all components, Figure S3: (a) Assignment of earphone plug-in: L = left channel, R = right channel, GND = ground, MIC = microphone and (b) Finished circuit board already connected to LED and battery clip and fixed in the add-on. Image courtesy (a) Produced with MS Visio by Christopher John and (b) Anna Friedrichs, Figure S4: All elements printed by the 3D printer: (a) Smart phone holding element, (b) Cuvette and electronics holding element, (c) Lid for electronics part to fix with screws, (d) Lid for cuvette, (e) Cover for LEDs, (f) The way to combine the lid for the cuvette and the cover of the LEDs, and (g) Filter set up. Image courtesy Anna Friedrichs, Figure S5: Additional material and already prepared elements: (a) Circuit board, (b) LED set up with one blue LED (and the option for a second one, grey square), (c) red transparency filter, (d) mirror of modelling and (e) Combination of circuit board and one connected LED already fitted into the add-on. Images courtesy Anna Friedrichs, Figure S6: Schematic view of the LED set up: aluminium plate (white) and breadboard (orange). (a) Side view of the LED set up, (b) Front view (including holes for screws), and (c) Front view with glued LED (including light cone), Figure S7: Image-by-image instruction to build the smart phone add-on to measure algal fluorescence in water: How (a) to glue the filter set up, (b) to position the mirror, (c) to fix the LED set up in to the 3D-element, (d) to place the cables, (e) to position the circuit board, (f) to combine the smart phone holding element (dark) and the prepared cuvette and electronics element (shadowed), (g) to place the lid of the electronics and (h) to place the lid of the cuvette and LED. Images (d), and (f) produced with e-drawings. Images (a), (b), (c), (e), (g), and (h) courtesy Anna Friedrichs, Figure S8: Completed SmartFluo-Switch, Figure S9: Circuit diagram of the LED control board including suggested connection points for battery-clip cable and earphone plug-in cable. AC- voltage amplifier is needed to amplify the earphone signal. Simulation of a sine is replaced by the frequency given through the earphone plug-in. Circuit set up is for a 100mA LED-current. Produced with LTSpice by Christopher John, Figure S10: Circuit board with all components in double layout for the possibility of two LEDs a) Top view (assembly side of the board) and b) Bottom side with soldering overview (soldering side of the board). Image courtesy Christopher John, Figure S11: (a) Assignment of earphone plug-in: L = left channel, R = right channel, GND = ground, MIC = microphone and (b) Finished circuit board including connections to LED, battery clip and earphone plug-in. Image courtesy (a) Produced with MS Visio by Christopher John and (b) Anna Friedrichs, Figure S12: All elements printed by the 3D printer: (a) Smart phone holding element, (b) Cuvette and electronics holding element, (c) Lid for electronics part to fix with screws, (d) Lid for cuvette, (e) Cover for LEDs, (f) The way to combine the lid for the cuvette and the cover of the LEDs, and (g) Filter set up. Image courtesy Anna Friedrichs, Figure S13: Additional material and already prepared elements: (a) Circuit board, (b) LED set up with one blue LED (and the option for a second one, grey square), (c) red transparency filter, (d) mirror of modelling and (e) Combination of circuit board, earphone plug-in and one connected LED. Image (a) Courtesy Christopher John, (b–e) Courtesy Anna Friedrichs, Figure S14: Schematic view of the LED set up: aluminium plate (white) and breadboard (orange). (a) Side view of the LED set up, (b) Front view (including holes for screws), and (c) Front view with glued LED (including light cone), Figure S15: Image-by-image instruction to build the smart phone add-on to measure algal fluorescence in water: How (a) to glue the filter set up, (b) to position the mirror, (c) to fix the LED set up in to the 3D-element, (d) to place the cables, (e) to position the circuit board, (f) to combine the smart phone holding element (dark) and the prepared cuvette and electronics element (shadowed), (g) to place the lid of the electronics and h) to place the lid of the cuvette and LED. Images (d), (e), and (f) produced with e-drawings. Images (a), (b), (c), (g), and (h) courtesy Anna Friedrichs, Figure S16: Completed SmartFluo-APP.

## References

[1] Cullen J.J. (1982). The Deep Chlorophyll Maximum: Comparing Vertical Profiles of Chlorophyll a. Can. J. Fish. Aquat. Sci..

[2] Klemas V. (2012). Remote Sensing of Algal Blooms: An Overview with Case Studies. J. Coast. Res..

[3] Behrenfeld M.J., O’Malley R.T., Siegel D.A., McClain C.R., Sarmiento J.L., Feldman G.C., Milligan A.J., Falkowski P.G., Letelier R.M., Boss E.S. (2006). Climate-driven trends in contemporary ocean productivity. Nat. Lett..

[4] Busch J.A., Zielinski O., Cembella A.D. (2013). Optical assessment of harmful algal blooms (HABs). Subsea Optics and Imaging.

[5] European Union Marine Strategy Framework Directive. http://ec.europa.eu.

[6] Govindjee, Papageorgiou G.C., Rabinowitch E. (1967). Chlorophyll fluorescence and photosynthesis. Fluorescence: Theory, Instrumentation, and Practice.

[7] Papageorgiou G. (1975). Chlorophyll fluorescence: An intrinsic probe of photosynthesis. Bioenergetics of Photosynthesis, Cell Biology.

[8] Krause G.H., Weis E. (1991). Chlorophyll fluorescence and photosynthesis: The Basics. Annu. Rev. Plant Physiol. Plant Mol. Biol..

[9] Govindjee (1995). Sixty-three years since Kautsky: Chlorophyll *a* fluorescence. Aust. J. Plant Physiol..

[10] Lakowicz J.R. (2010). Principles of Fluorescence Spectroscopy.

[11] Huot Y., Brown C.A., Cullen J.J. (2005). New algorithms for MODIS sun-induced chlorophyll fluorescence and a comparison with present data products. Limnol. Oceanogr. Methods.

[12] Behrenfeld M.J., Westberry T.K., Boss E.S., O’Malley R.T., Siegel D.A., Wiggerta J.D., Franz B.A., McClain C.R., Feldman G.C., Doney S.C. (2009). Satellite-detected fluorescence reveals global physiology of ocean phytoplankton. Biogeoscience.

[13] Kirk J.T.O. (2011). Light and Photosynthesis in Aquatic Ecosystems.

[14] Maxwell K., Johnson G.N. (2000). Chlorophyll fluorescence—A practical guide. J. Exp. Bot..

[15] Brunet C., Johnsen G., Lavaud J., Roy S. (2011). Pigments and photoacclimation processes. Phytoplankton Pigments: Characterization, Chemotaxonomy and Applications in Oceanography.

[16] Lorenzen C.J. (1966). A method for the continuous measurements of in vivo chlorophyll concentration. Deep-Sea Res..

[17] Babin M. (2008). Phytoplankton fluorescence: Theory, current literature and in situ measurements. Real-time Coastal Observing Systems for Marine Ecosystem Dynamics and Harmful Algal Blooms.

[18] Moore C., Barnard A., Fietzek P., Lewis M., Sosik H., White S., Zielinski O. (2009). Optical tools for ocean monitoring and research. Ocean Sci..

[19] Pires M.D. (2010). Evaluation of Fluorometers for the in situ Monitoring of Chlorophyll and/or Cyanobacteria.

[20] Zielinski O., Busch J.A., Cembella A.D., Daly K.L., Engelbrektsson J., Hannides A.K., Schmidt H. (2009). Detecting marine hazardous substances and organisms: Sensors for pollutants, toxins, and pathogens. Ocean Sci..

[21] Johnsen G., Moline M.M., Pettersson L.H., Pinckney J., Pozdnyakoy D.V., Egeland E.S., Schofield O.M. (2011). Optical monitoring of phytoplankton bloom pigment signatures. Phytoplankton Pigments Characterization, Chemotaxonomy and Applications in Oceanography.

[22] Chang G.C., Dickey T.D. (2008). Interdisciplinary sampling strategies for detection and characterization of harmful algal blooms. Real-Time Coastal Observing Systems for Marine Ecosystems Dynamics and Harmful Algal Blooms.

[23] Busch J.A., Price I., Jeansou E., Zielinski O., van der Woerd H.J. (2016). Citizens and satellites: Assessment of phytoplankton dynamics in a NW Mediterranean aquaculture zone. Int. J. Appl. Earth Obs. Geoinf..

[24] Leeuw T., Boss E.S., Wright D.L. (2013). In situ measurements of phytoplankton fluorescence using low-cost electronics. Sensors.

[25] CITCLOPS. (Citizens’ observatory for Coast and Ocean Optical Monitoring). www.citclops.eu.

[26] Novoa S., Wernand M.R., van der Woerd H.J. (2015). WACODI: A generic algorithm to derive the intrinsic color of natural waters from digital images. Limnol. Oceanogr. Methods.

[27] Wright W.D. (1929). A re-determination of the trichromatic coefficients of the spectral colours. Trans. Opt. Soc..

[28] Smith T., Guild J. (1931). The C.I.E. colorimetric standards and their use. Trans. Opt. Soc..

[29] Fairman H.S., Brill M.H., Hemmendinger H. (1997). How the CIE 1931 color-matching functions were derived from Wright-Guild data. Color Res. Appl..

[30] Cullen J.J., Davis R.F. (2003). The blank can make a big difference in oceanographic measurements. Limnol. Oceanogr. Bull..

[31] Arar E.J., Collins G.B. (1997). EPA-method 445.0, In vitro Determination of Chlorophyll a and Pheophytin a in Marine and Freshwater Algae by Fluorescence. Methods for Determination of Chemical Substances in Marine and Estuarine Matrices.

[32] Wernand M.R., van der Woerd H.J. (2010). Spectral analysis of the Forel-Ule Ocean colour comparator scale. J. Eur. Opt. Soc. Rapid Publ..

[33] Novoa S., Wernand M.R., van der Woerd H.J. (2014). The modern Forel-Ule scale: A ‘do-it-yourself’ colour comparator for water monitoring. J. Eur. Opt. Soc. Rapid Publ..

[34] White M., Feighery L., Bowers D., O’Riain G., Bowyer P. (2005). Using digital cameras for river plume and water quality measurements. Int. J. Remote Sens..

[35] Kuo Y.S., Verma S., Schmid T., Dutta P. Hijacking power and bandwidth from the mobile phone’s audio interface. Proceedings of the First ACM Symposium on Computing for Development.

[36] Johnsen G., Bricaud A., Nelson N., Prèzelin B.B., Bidigare R.R. (2011). In vivo bio-optical properties of phytoplankton pigments. Phytoplankton Pigments Characterization, Chemotaxonomy and Applications in Oceanography.

[37] Welschmeyer N.A. (1994). Fluorometric analysis of chlorophyll a in the presence of chlorophyll b and pheopigments. Limnol. Oceanogr..

[38] Busch J.A., Bardaji R., Ceccaroni L., Friedrichs A., Piera J., Simon C., Thijsse P., Wernand M.R., van der Woerd H.J., Zielinski O. (2016). Citizen Bio-Optical Observations from Coast- and Ocean and Their Compatibility with Ocean Colour Satellite Measurements. Remote Sens..

[39] Fienen M.N., Lowry C.S. (2012). Social.Water—A crowdsourcing tool for environmental data acquisition. Comput. Geosci..

[40] Eyeonwater.Org Colour. www.eyeonwater.org/color.

[41] Tulloch A.I., Possingham H.P., Joseph L.N., Szabo J., Martin T.G. (2013). Realising the full potential of citizen science monitoring programs. Biol. Conserv..

[42] Zielinski O. (2013). Subsea optics: An introduction. Subsea Optics and Imaging.

[43] Beutler M., Wiltshire K., Meyer B., Moldaenke C., Lüring C., Meyerhöfer M., Hansen U.P., Dau H. (2002). A fluorometric method for the differentiation of algal populations in vivo and in situ. Photosynth. Res..

