# Supplementary material for: SmartFluo: A Method and Affordable Adapter to Measure Chlorophyll a Fluorescence with Smartphones
Source: Sensors (Basel). 2017 Mar 25;17(4):678. doi: 10.3390/s17040678 (PMC5419791; doi:10.3390/s17040678)
Supplement: Supplementary file 1 [file sensors-17-00678-s001.pdf]

# SmartFluo >> How to build the smart phone add-on to measure algal fluorescence in water <<

**New developments in this instrument will be published on the website: www.citclops.eu. Therefore, we recommend to check the web-based instruction first, before building the SmartFluo.**

**Table of content**


README 1

PART 1 – 3D print 2

PART 2 – A) Electronics for SmartFluo-Switch 3

PART 2 – B) Assembling for SmartFluo-Switch 5

PART 3 – A) Electronics for SmartFluo-APP 9

PART 3 – B) Assembling for SmartFluo-APP 11

Algorithm for interpretation of the fluorescence images 14


## README

SmartFluo is a smart phone add-on to measure algal fluorescence in water. With the SmartFluo you do not have to dip your smart phone into the water. But you can still measure the algal fluorescence by means of a small add-on, some additional material, and a cuvette. In the following, I will explain how to build such a smart phone add-on. You already know fluorescence from the webpage (www.citclops.eu) or you learnt it at school or university, so I will not delve into this topic. At the webpage you also find a video and step-by-step instruction on how to use the SmartFluo to measure algal fluorescence in water. Learn more about the calculation of fluorescence on our webpage:
http://www.citclops.eu/fluorescence-/measuring-water-fluorescence

Keep an eye on water quality! Have fun!

Currently the 3D files for printing are available only for the Samsung Galaxy S3 mini, but you are welcome to adapt and share new files for other smart phone types.
Before we start to build the add-on, find below a table with all elements you need, including suggested suppliers. SmartFluo is available in switch-version (SmartFluo-Switch; suitable for beginners in electronics) and APP-version (SmartFluo-APP; suitable for experts in electronics). You will find build instruction for the switch-version starting at page 3 and for the APP-version starting at page 9. You will find a list of required material in Table 1 at page 2.

Building the add-on has three major steps separated in several minor steps:

(1) All 3D elements are printed with a 3D printer, see PART 1 – 3D print
(2) Building the electronics for either the switch- or APP-version, see PART 2 – A) Electronics or PART 3 – A) Electronics
(3) Assembling 3D elements and the electronics for either the switch- or APP-version, see PART 2 – B) Assembling or PART 3 – B) Assembling

This instruction was produced within Citclops. The smart phone add-on was developed and built by the members of the project partner University of Oldenburg, Institute for Chemistry and Biology of the Marine Environment, working group Marine Sensor Systems. Citclops is supported by the EC-FP7 Programme, grant agreement nº 308469. We thank EU for funding!

# SmartFluo >> How to build the smart phone add-on to measure algal fluorescence in water <<

**Table 1:** *All components you need with costs and suggested suppliers at a glance; [a] taken from a filter booklet; [b]sales quantity unit: 100; [c] sold in 200×30×5 mm (l×w×h); [d]price depends on provider. White: Material for both versions, blue: additional for switch-version, green: additional for APP-version.*

| Component | Suggested Supplier | Price [€] |
|---|---|---|
| Smart phone: Galaxy S3 mini | Samsung Electronics, South Korea | - |
| Transparency filters[a] red #19 | Roscolux, CT, USA | 10.97 |
| Mini mirror (modeling) | K & L Bürotechnik GmbH, Germany | 1,81 |
| Cuvette[b] | Brand, Germany | 0.11 |
| Aluminium plate[c] | Conrad Electronic SE, Germany | 6.49 |
| 3D-printed elements | different | ~ 25[d] |
| Screws (small) | HORNBACH Holding AG, Germany | < 2 |
| High-Power-LED (448 nm) | Philips Lumileds Lighting Company, CA, USA | 5.00 |
| Nine-volt battery including a clip | VARTA Consumer Batteries, Germany | 1.92 |
| 3,5mm phone plug-in (four poles) | Lumberg Holding, Germany | 0.30 |
| Voltage regulator LM317 | National Semiconductor, TX, USA | 0.82 |
| Resistor (various) | Yageo Corporation, Taiwan | 0.45 |
| Two-core cable | JAMARA e.K., Germany | 1.70 |
| Breadboard | RADEMACHER Geräte-Elektronik, Germany | 0.85 |
| Midget toggle switch three poles | Conrad Electronic SE, Germany | ~ 3 |
| Film Capacitor (various) | WIMA, Germany | 0.88 |
| Transistor | Micro Electronics Ltd., Hong Kong | 0.68 |
| Diode | STMicroelectronics N.V., Switzerland | 0.04 |

## PART 1 – 3D print

### List of STL files for 3D printer in the ZIP-folder Citclops_SmartFluo_STLfilesfor3Dprint

1) 01_Citclops_SamsungGalaxysiiiminiholder.stl
2) 02_Citclops_CuvettenholderandexternalLED.stl
3) 03_Citclops_Lidforcuvette.stl
4) 04_Citclops_Lidforelektronic.stl
5) 05_Citclops_CoverforexternalLED.stl
6) 06_Citclops_Filtersetup.stl

For building the SmartFluo you need to print each of the named files above once. When you have finished the 3D prints for SmartFluo-Switch go to PART 2 – A) Electronics for SmartFluo-Switch and for SmartFluo-APP go to PART 3 – A) Electronics for SmartFluo-APP.

HINT: Sometimes it is necessary to enlarge a hole while building the smart phone add-on e.g. the hole for the cables. In most cases you have to sand the filter set up in the end until it fits into the slit.

This instruction was produced within Citclops. The smart phone add-on was developed and built by the members of the project partner University of Oldenburg, Institute for Chemistry and Biology of the Marine Environment, working group Marine Sensor Systems. Citclops is supported by the EC-FP7 Programme, grant agreement nº 308469. We thank EU for funding!

# SmartFluo >> How to build the smart phone add-on to measure algal fluorescence in water <<

## PART 2 – A) Electronics for SmartFluo-Switch

Building the electronics for SmartFluo-Switch requires only a little knowledge of electronics.

Keep an eye on water quality! Have fun!

### Material for preparing the electronic part (for 100mA LED-current)

**Table 2:** *List of material for set up electric of SmartFluo-Switch: with specifications and used abbreviation for circuit board.*

| Amount | Specification | Component | Abbreviation, Fig. 1 |
|---|---|---|---|
| 1 | 12Ω | Resistor | R1 |
| 1 | LM317 | Voltage regulator | ADJ |
| 1 | (KNX-1) three poles | Midget toggle switch | S1 |
| 1 | 448 nm | High-Power-LED | LED1 |
| 1 | | Nine-volt battery including a clip | G1 |
| 1 | 3,5mm (four poles) | phone plug-in | |
| 1m | | Two-core cable | |
| 1 | 50×100 mm (l × w) | Breadboard with pads | |

### Building instruction

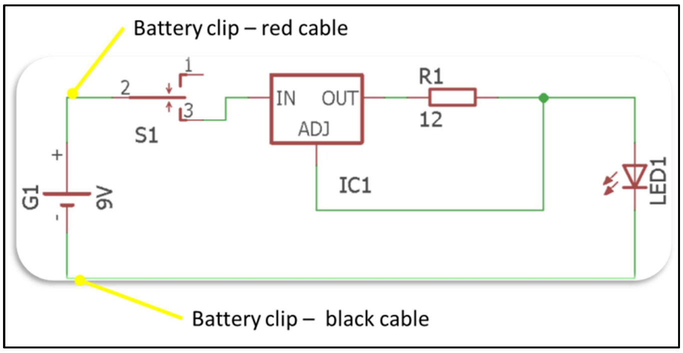


**Fig. 1:** *Circuit diagram of the LED control board including suggested connection points for battery-clip cable. Circuit set up is for a 100mA LED-current (IC1). Produced with EAGLE CadSoft by Rohan Henkel.*

Set up the entire circuit for one LED based on the circuit diagram, shown in Fig. 1, with the following steps:

1) Leave the breadboard (with copper pads) initially at full size. It can be brought later by scribing and breaking at one edge to a smaller size. The board size required is at most 53x36 mm.
2) First fit the voltage regulator and add the resistor and then add the solder to them, see Fig. 2. Pinch off any excess with a wire cutter.

This instruction was produced within Citclops. The smart phone add-on was developed and built by the members of the project partner University of Oldenburg, Institute for Chemistry and Biology of the Marine Environment, working group Marine Sensor Systems. Citclops is supported by the EC-FP7 Programme, grant agreement nº 308469. We thank EU for funding!

# **SmartFluo** >> How to build the smart phone add-on to measure algal fluorescence in water <<

3) In the last step, assemble the connections to the LED, to the 9V block battery (with a clip) and to the switch (see Fig. 2). HINT: the left and the right channel and GND=ground must be assigned, see Fig. 3a. Over the right channel one LED is driven and over the left channel another LED can be driven. Also an additional hole in the upper part of the add-on is needed to fix and use the switch (Fig. 3b).

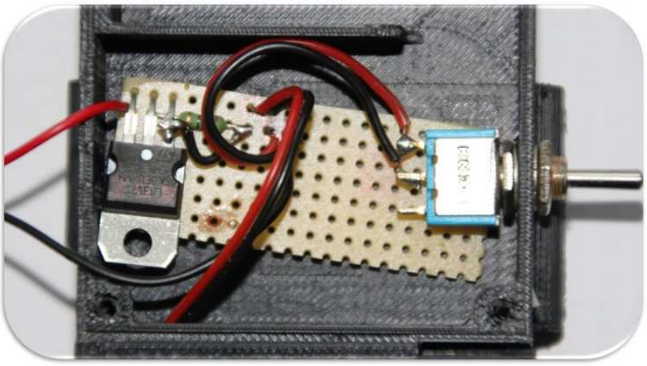

**Fig. 2:** *Circuit board with all components*

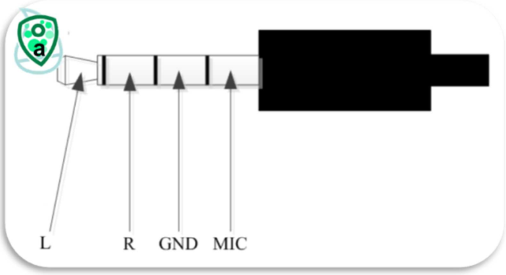


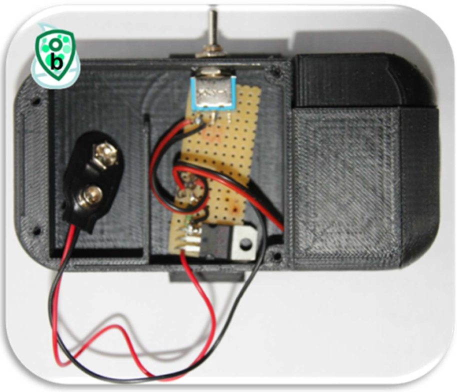


**Fig. 3:** *a) Assignment of earphone plug-in: L = left channel, R = right channel, GND = ground, MIC = microphone and b) Finished circuit board already connected to LED and battery clip and fixed in the add-on. Image courtesy a) Produced with MS Visio by Christopher John and b) Anna Friedrichs.*

This instruction was produced within Citclops. The smart phone add-on was developed and built by the members of the project partner University of Oldenburg, Institute for Chemistry and Biology of the Marine Environment, working group Marine Sensor Systems. Citclops is supported by the EC-FP7 Programme, grant agreement nº 308469. We thank EU for funding!

# SmartFluo >> How to build the smart phone add-on to measure algal fluorescence in water <<

## PART 2 – B) Assembling the SmartFluo-Switch

Assembling the SmartFluo-Switch is the combination of the electronic part with the 3D elements and includes the preparation of the LED set up.

Keep an eye on water quality! Have fun!

### Material from the 3D printer

1) Smart phone holding element (Fig. 4a)
2) Cuvette and electronics holding element (Fig. 4b)
3) Lid for electronic part (Fig. 4c)
4) Lid for cuvette (Fig. 4d)
5) Cover for LEDs (Fig. 4e)
6) Filter set up (Fig. 4g)

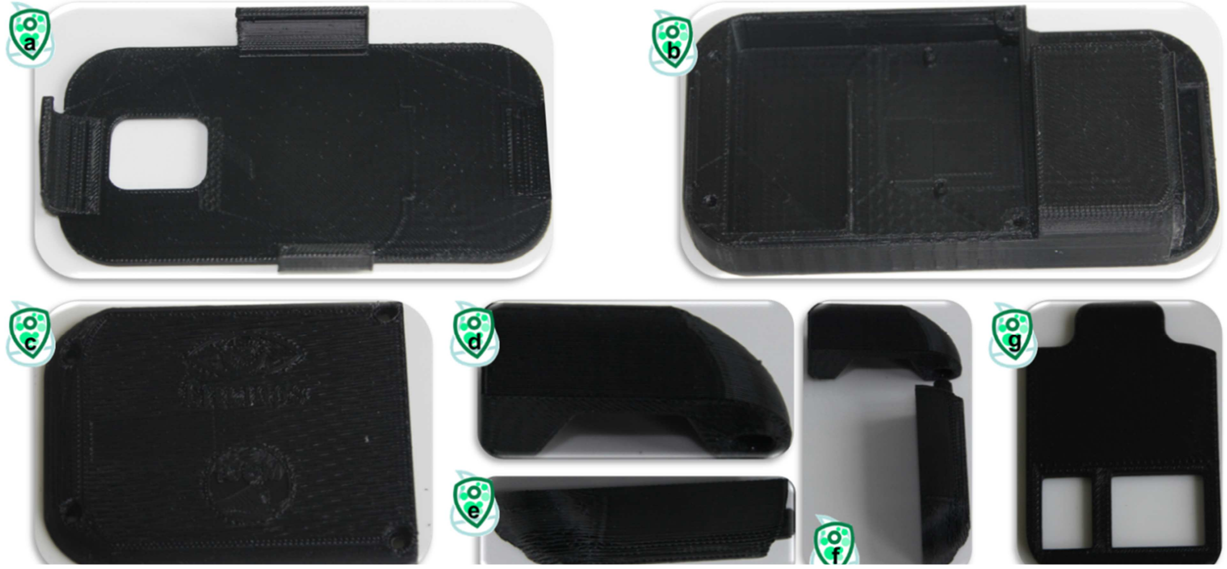


**Fig. 4:** *All elements printed by the 3D printer: a) Smart phone holding element, b) Cuvette and electronics holding element, c) Lid for electronics part to fix with screws, d) Lid for cuvette, e) Cover for LEDs, f) The way to combine the lid for the cuvette and the cover of the LEDs, and g) Filter set up. Image courtesy Anna Friedrichs.*

If you have the 3D elements at hand, then add the additional material as given below. Now I will explain how to combine all elements to the SmartFluo-Switch.

### Additional material and prepared electronic elements

1) 9V Battery (not shown)
2) Circuit board (Fig. 5a, see PART 2 – A) Electronics for SmartFluo-Switch)
3) LED set up (Fig. 5b, see Build instruction for the LED set up)
4) Filter (Fig. 5c, red #19)
5) Mirror (Fig. 5d)
6) Aluminium plate (15×8×5 mm, l × w × h)
7) Screws

This instruction was produced within Citclops. The smart phone add-on was developed and built by the members of the project partner University of Oldenburg, Institute for Chemistry and Biology of the Marine Environment, working group Marine Sensor Systems. Citclops is supported by the EC-FP7 Programme, grant agreement nº 308469. We thank EU for funding!

# SmartFluo >> How to build the smart phone add-on to measure algal fluorescence in water <<

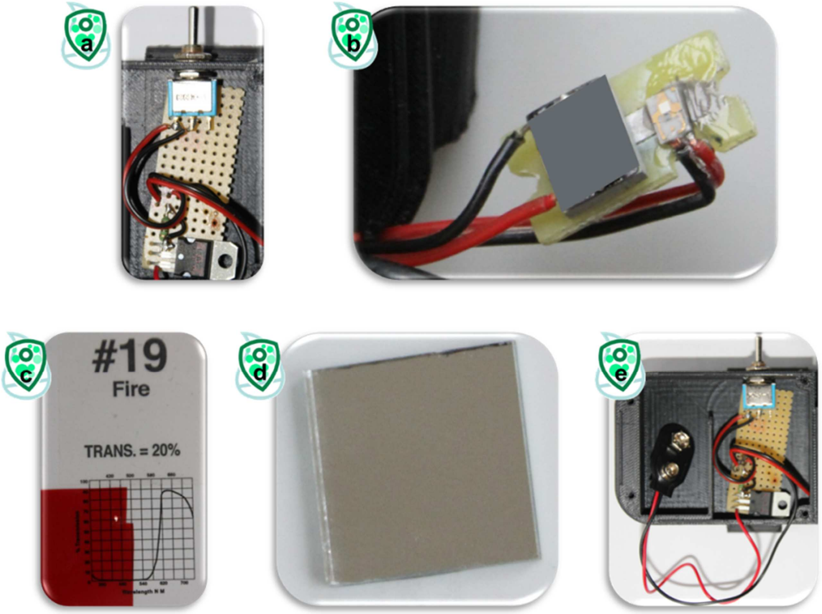


**Fig. 5:** *Additional material and already prepared elements: a) Circuit board, b) LED set up with one blue LED (and the option for a second one, grey square), c) red transparency filter, d) mirror of modelling and e) Combination of circuit board and one connected LED already fitted into the add-on. Images courtesy Anna Friedrichs.*

### Build instruction for the LED set up

1) Take a piece (22 mm x 11 mm) of the already available breadboard and remove all copper pads.
2) Enter a hole with the size of 15 mm x 5 mm in the middle of the piece.
3) Glue the aluminium plate within the hole (see Fig. 6a&b)
4) Glue the LED with thermally conductive glue onto this prepared setup (see Fig. 6c).

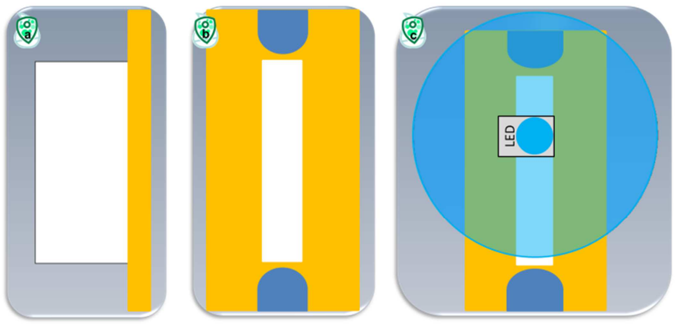


**Fig. 6:** *Schematic view of the LED set up: aluminium plate (white) and breadboard (orange). a) Side view of the LED set up, b) Front view (including holes for screws), and c) Front view with glued LED (including light cone).*

HINT: If you fix the LED set up within the add-on, you might have to arrange the cables of the LED in an additional hole in the breadboard (see Fig. 5b and Fig. 7c).

This instruction was produced within Citclops. The smart phone add-on was developed and built by the members of the project partner University of Oldenburg, Institute for Chemistry and Biology of the Marine Environment, working group Marine Sensor Systems. Citclops is supported by the EC-FP7 Programme, grant agreement nº 308469. We thank EU for funding!

# SmartFluo >> How to build the smart phone add-on to measure algal fluorescence in water <<

Now you prepared all additional material and the electronics (Fig. 5e) including the LED set up which you need to build the SmartFluo-Switch.

### Construction guidance SmartFluo-Switch

1) Glue the red filter into the filter set up. Take care of the position of the red filter (Fig. 7a)! You might have to sand it.
2) Glue the mirror into the ‘cuvette and electronics holding element’ (Fig. 7b).
3) Add a hole at the top side of the add-on (see Fig. 7a) to fix the toggle switch (Fig. 7e).
4) Screw the LED set up into the ‘cuvette and electronics holding element’ (Fig. 7c).
5) Thread the cable of the LED set up through the hole of the ‘cuvette and electronics holding element’ to the place of the circuit board (Fig. 7d). You might have to enlarge the hole.
6) Fix the circuit board at allocated space with screws (Fig. 7e).
7) Glue the now prepared ‘cuvette and electronics holding element’ onto the ‘smart phone holding element’ with glue (Fig. 7f).
8) Add the 9V-block battery and fix the lid of the electronics part with screws (Fig. 7g).
9) Add the cover of the LEDs and the lid of the cuvette (Fig. 7h). HINT: Glue the cover of the LEDs at the end.

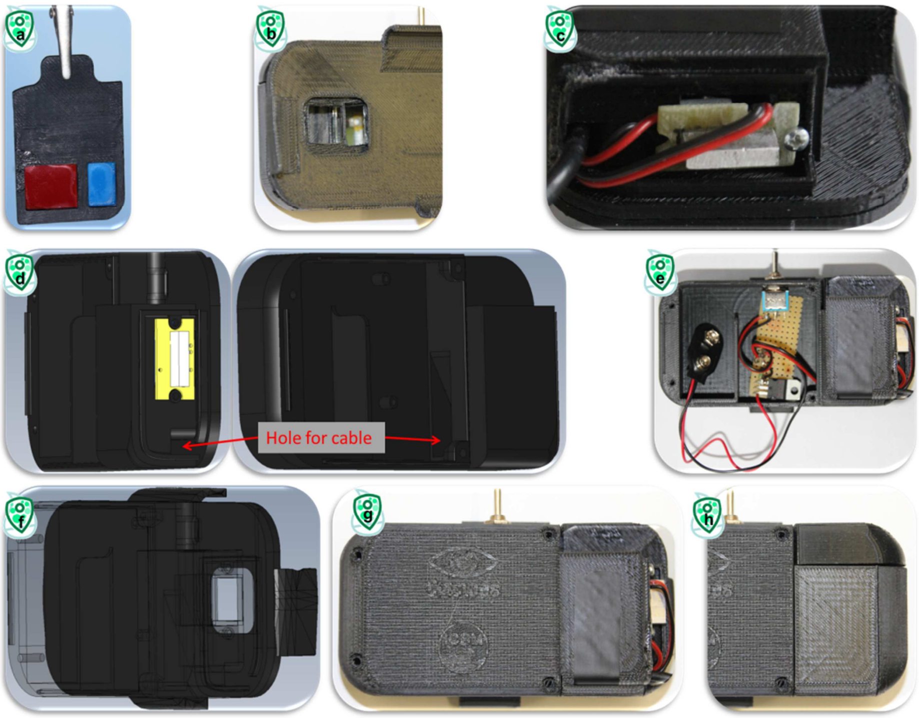


**Fig. 7:** *Image-by-image instruction to build the smart phone add-on to measure algal fluorescence in water: How a) to glue the filter set up, b) to position the mirror, c) to fix the LED set up in to the 3D-element, d) to place the cables, e) to position the circuit board, f) to combine the smart phone holding element (dark) and the prepared cuvette and electronics element (shadowed), g) to place the lid of the electronics and h) to place the lid of the cuvette and LED. Images d), and f) produced with e-drawings. Images a), b), c), e), g), and h) courtesy Anna Friedrichs.*

This instruction was produced within Citclops. The smart phone add-on was developed and built by the members of the project partner University of Oldenburg, Institute for Chemistry and Biology of the Marine Environment, working group Marine Sensor Systems. Citclops is supported by the EC-FP7 Programme, grant agreement nº 308469. We thank EU for funding!

# SmartFluo >> How to build the smart phone add-on to measure algal fluorescence in water <<

You are done! Now you just combine the add-on with the smart phone; it should look like Figure 8. You hold a powerful fluorescence sensor system in your hands!

**DONE!**

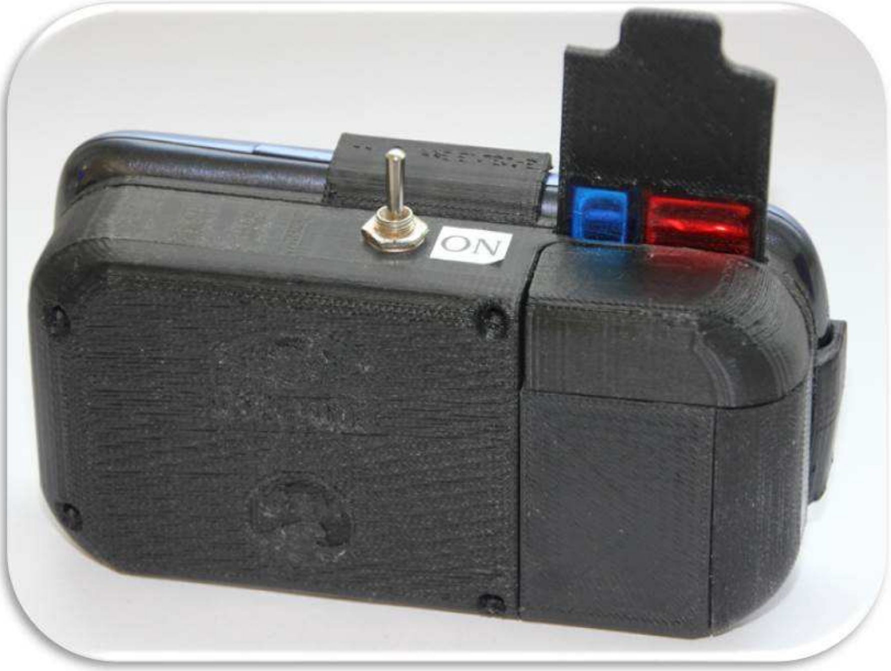


**Fig. 8:** Completed SmartFluo-Switch

**To share your data, please prepare a metadata form manually and send form and images to us. Contact person: oliver.zielinski@uni-oldenburg.de**

**For data download use: http://www.citclops.eu/search/welcome.php**

**Thank you for your contribution!**

This instruction was produced within Citclops. The smart phone add-on was developed and built by the members of the project partner University of Oldenburg, Institute for Chemistry and Biology of the Marine Environment, working group Marine Sensor Systems. Citclops is supported by the EC-FP7 Programme, grant agreement nº 308469. We thank EU for funding!

# SmartFluo >> How to build the smart phone add-on to measure algal fluorescence in water <<

## PART 3 – A) Electronics for SmartFluo-APP

Building the electronics for SmartFluo-APP requires some knowledge of electronics.

Keep an eye on water quality! Have fun!

### Material for preparing the electronic part (for 100mA LED-current)

**Table 3:** *List of material for set up electric of SmartFluo-APP: with specifications and used abbreviation for circuit board.*

| Amount | Specification | Component | Abbreviation, Fig. 9 |
|---|---|---|---|
| 3<br>1 | 100nF<br>470nF, 2.2µF | Film Capacitor | C2, C4, C5<br>C3, C1 |
| 1<br>1 | 22kΩ, 10kΩ, 470Ω,<br>200Ω, 100Ω, 82Ω, 12Ω | Resistor | R1, R2, R3,<br>R4, R5, R7, R6 |
| 2 | 2N2222 (TO-18 housing) | Transistor | Q1, Q2 |
| 1 | LM317 (SOT-223 housing) | Voltage regulator | ADJ |
| 2 | 1N4148 | Diode | D1, D2 |
| 1 | 448 nm | High-Power-LED | D3 (LXHL-BW02) |
| 1 | | Nine-volt battery including a clip | V2 |
| 1 | 3,5mm (four poles) | phone plug-in | |
| 1m | | Two-core cable | |
| 1 | 50×100 mm (l × w) | Breadboard with pads | |

### Building instruction

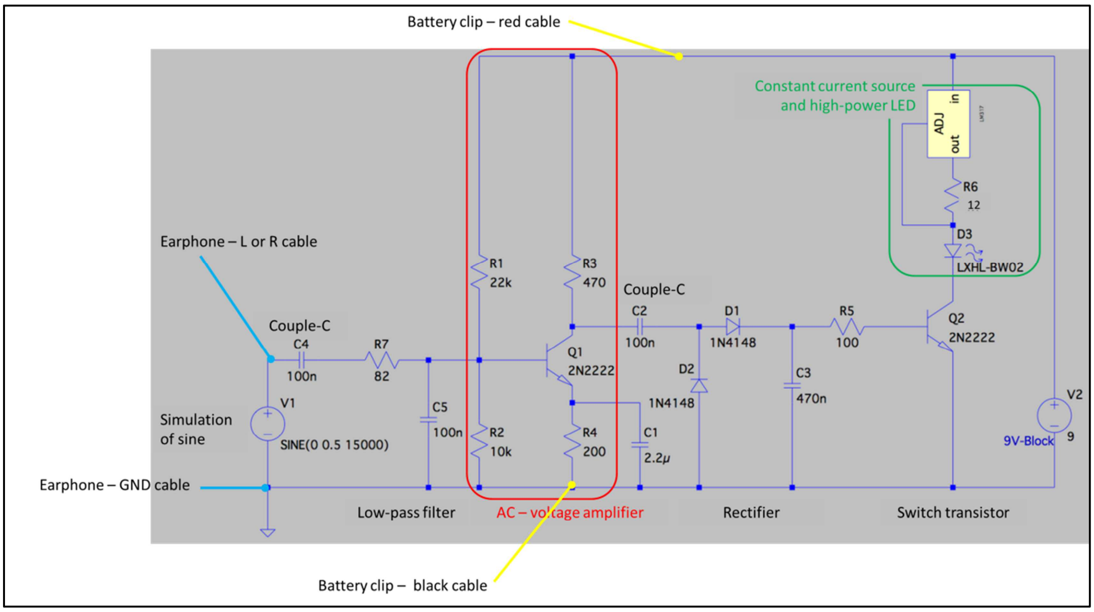


**Fig. 9:** *Circuit diagram of the LED control board including suggested connection points for battery-clip cable and earphone plug-in cable. AC- voltage amplifier is needed to amplify the earphone signal. Simulation of a sine is replaced by the frequency given through the earphone plug-in. Circuit set up is for a 100mA LED-current. Produced with LTSpice by Christopher John.*

This instruction was produced within Citclops. The smart phone add-on was developed and built by the members of the project partner University of Oldenburg, Institute for Chemistry and Biology of the Marine Environment, working group Marine Sensor Systems. Citclops is supported by the EC-FP7 Programme, grant agreement nº 308469. We thank EU for funding!

# SmartFluo >> How to build the smart phone add-on to measure algal fluorescence in water <<

Set up the entire circuit for one LED based on the circuit diagram, shown in Fig. 9, with the following steps:

1) Leave the breadboard (with copper pads) initially at full size. It can be adjusted later by scribing and breaking at one edge to a smaller size. The board size required is 53x36 mm.
2) First fit all flat components such as resistors and diodes. After insertion of the component in each case the legs are bending over, so the component cannot fall off. Then you solder them (Fig. 10b). Pinch off any excess with a wire cutter. HINT: The resistors can also be fitted upright for a smaller footprint, see Fig. 10a; The LM317 is soldered on the bottom side.
3) Continue with insertion of the higher components, e.g. the 2.2µF capacitors. HINT: For the assignment of the connections of the 2N2222 transistors and the LM317 voltage regulators, take a look at the datasheets. Solder them, if all components are fitted, see Fig. 10a&b.
4) Assemble the necessary connections on the soldering side, see Fig. 10b. HINT: For longer connections silver wire can be used.
5) In the last step, assemble the connections to the LED, to the 9V block battery (with a clip) and to the 3.5mm phone plug-in with the two-core cable, see Fig. 10a&b. HINT: the left and the right channel and GND must be assigned, see Fig. 11a. Over the right channel one LED is driven and over the left channel another LED can be driven.

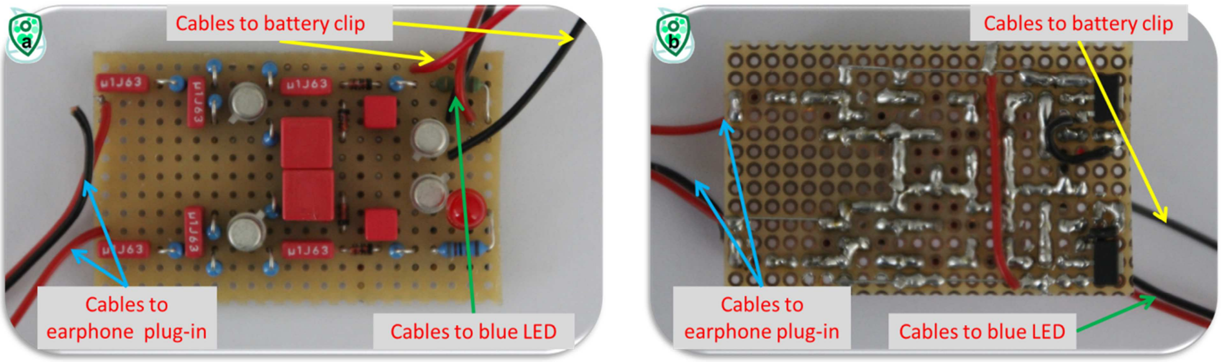


**Fig. 10:** *Circuit board with all components in double layout for the possibility of two LEDs a) Top view (assembly side of the board) and b) Bottom side with soldering overview (soldering side of the board). Image courtesy Christopher John.*

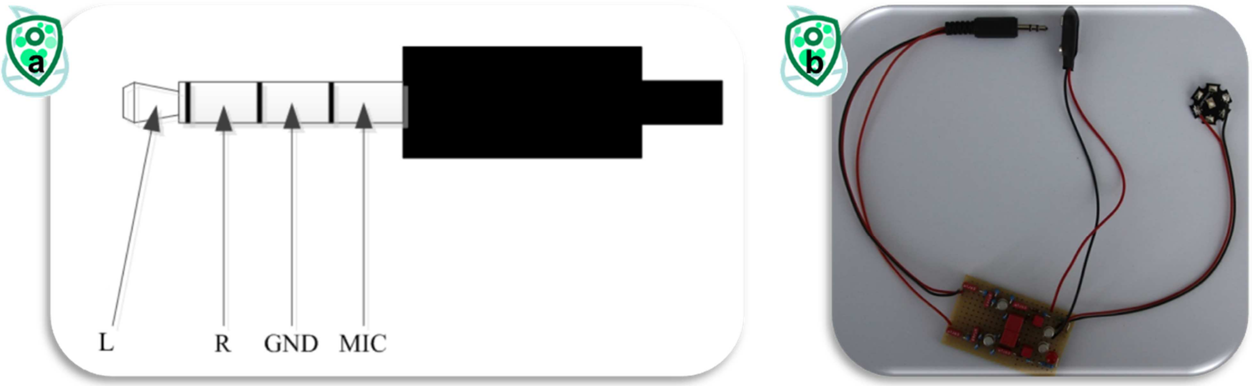


**Fig. 11:** *a) Assignment of earphone plug-in: L = left channel, R = right channel, GND = ground, MIC = microphone and b) Finished circuit board including connections to LED, battery clip and earphone plug-in. Image courtesy a) Produced with MS Visio by Christopher John and b) Anna Friedrichs.*

This instruction was produced within Citclops. The smart phone add-on was developed and built by the members of the project partner University of Oldenburg, Institute for Chemistry and Biology of the Marine Environment, working group Marine Sensor Systems. Citclops is supported by the EC-FP7 Programme, grant agreement nº 308469. We thank EU for funding!

# SmartFluo >> How to build the smart phone add-on to measure algal fluorescence in water <<

## PART 3 – B) Assembling for SmartFluo-APP

Assembling the SmartFluo-APP is the combination of the electronic part with the 3D elements and includes the preparation of the LED set up.

Keep an eye on water quality! Have fun!

**Material from the 3D printer**

1) Smart phone holding element (Fig. 12a)
2) Cuvette and electronics holding element (Fig. 12b)
3) Lid for electronic part (Fig. 12c)
4) Lid for cuvette (Fig. 12d)
5) Cover for LEDs (Fig. 12e)
6) Filter set up (Fig. 12g)

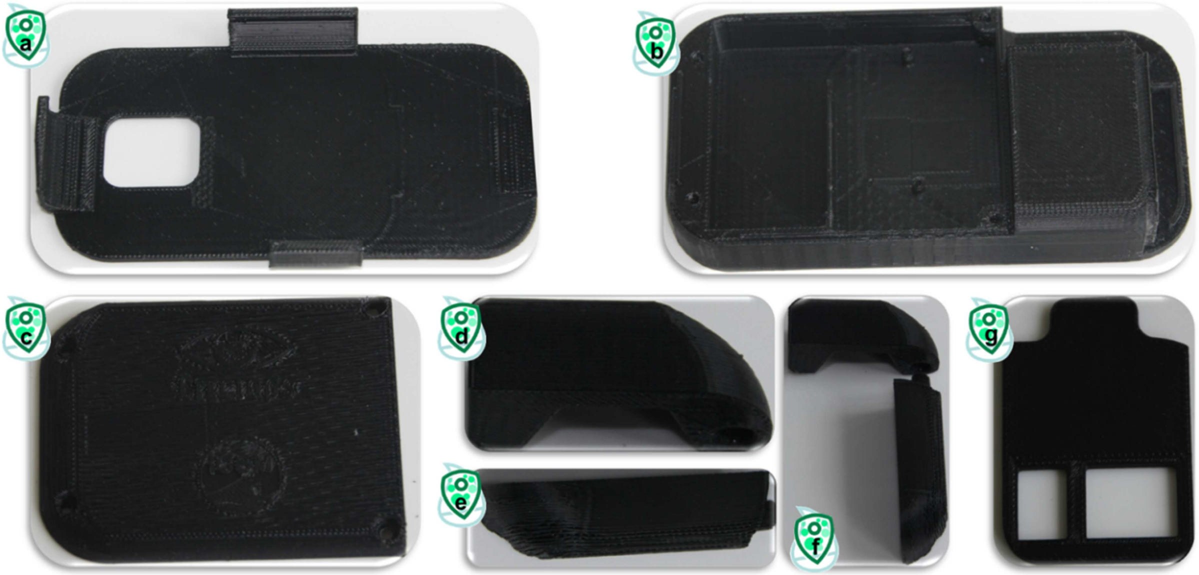

**Fig. 12:** *All elements printed by the 3D printer: a) Smart phone holding element, b) Cuvette and electronics holding element, c) Lid for electronics part to fix with screws, d) Lid for cuvette, e) Cover for LEDs, f) The way to combine the lid for the cuvette and the cover of the LEDs, and g) Filter set up. Image courtesy Anna Friedrichs.*

If you have the 3D elements at hand, then add the additional material as given in the following. Now I will explain how to combine all elements to the SmartFluo-APP.

**Additional material and prepared electronic elements**

1) 9V Battery (not shown)
2) Circuit board (Fig. 13a, see PART 3 – A) Electronics for SmartFluo-APP)
3) LED set up (Fig. 13b, see
4) Building instruction for the LED set up)
5) Filter (Fig. 13c, red #19)
6) Mirror (Fig. 13d)
7) Aluminium plate (15×8×5 mm, l × w × h)
8) Screws

This instruction was produced within Citclops. The smart phone add-on was developed and built by the members of the project partner University of Oldenburg, Institute for Chemistry and Biology of the Marine Environment, working group Marine Sensor Systems. Citclops is supported by the EC-FP7 Programme, grant agreement nº 308469. We thank EU for funding!

# SmartFluo >> How to build the smart phone add-on to measure algal fluorescence in water <<

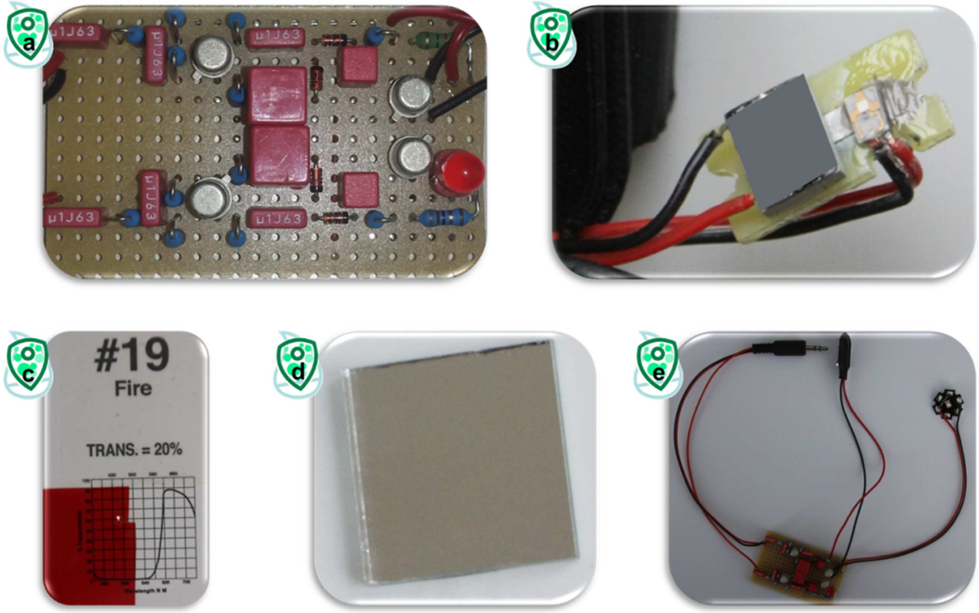


**Fig. 13:** *Additional material and already prepared elements: a) Circuit board, b) LED set up with one blue LED (and the option for a second one, grey square), c) red transparency filter, d) mirror of modelling and e) Combination of circuit board, earphone plug-in and one connected LED. Image a) Courtesy Christopher John, b-e) Courtesy Anna Friedrichs.*

## Building instruction for the LED set up

1) Take a piece (22 mm x 11 mm) of the already available breadboard and remove all copper pads.
2) Enter a hole with the size of 15 mm x 5 mm in the middle of the piece.
3) Glue the aluminium plate within the hole (see Fig. 14a&b).
4) Glue the LED with thermally conductive glue onto this prepared setup (see Fig. 14c).

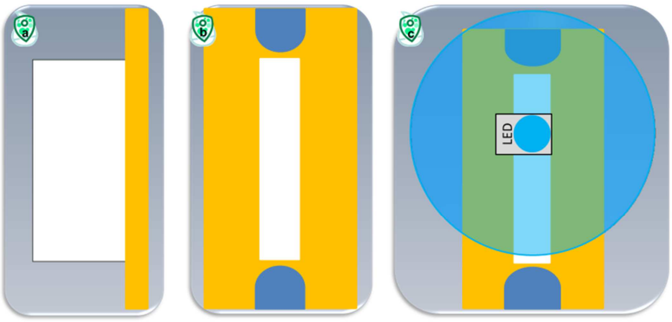


**Fig. 14:** *Schematic view of the LED set up: aluminium plate (white) and breadboard (orange). a) Side view of the LED set up, b) Front view (including holes for screws), and c) Front view with glued LED (including light cone).*

HINT: If you fix the LED set up within the add-on, you might have to arrange the cables of the LED in an additional hole in the breadboard (see Fig. 13b and Fig. 15c).

This instruction was produced within Citclops. The smart phone add-on was developed and built by the members of the project partner University of Oldenburg, Institute for Chemistry and Biology of the Marine Environment, working group Marine Sensor Systems. Citclops is supported by the EC-FP7 Programme, grant agreement nº 308469. We thank EU for funding!

# SmartFluo >> How to build the smart phone add-on to measure algal fluorescence in water <<

Now you prepared all additional material and the electronics (Fig. 13e) including the LED set up which you need to build the SmartFluo-APP.

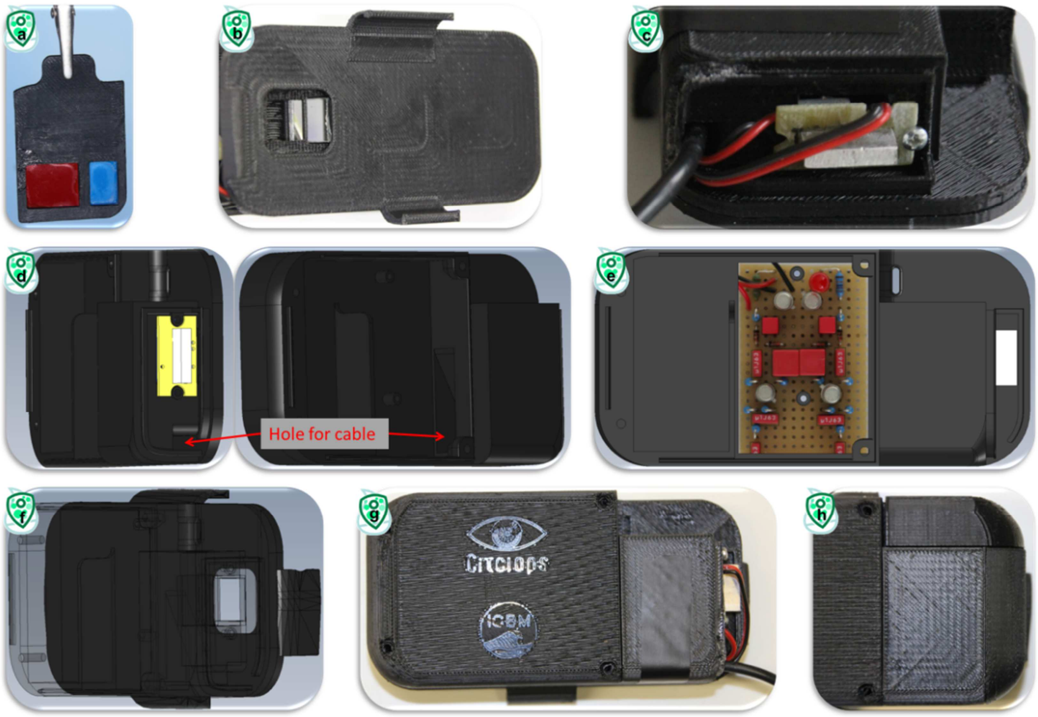


**Fig. 15:** *Image-by-image instruction to build the smart phone add-on to measure algal fluorescence in water: How a) to glue the filter set up, b) to position the mirror, c) to fix the LED set up in to the 3D-element, d) to place the cables, e) to position the circuit board, f) to combine the smart phone holding element (dark) and the prepared cuvette and electronics element (shadowed), g) to place the lid of the electronics and h) to place the lid of the cuvette and LED. Images d), e), and f) produced with e-drawings. Images a), b), c), g), and h) courtesy Anna Friedrichs.*

### Construction guidance SmartFluo-APP

1) Glue the red filter into the filter set up. Take care of the position of the red filter (Fig. 15a)! You might have to sand it at the end.
2) Glue the mirror into the ‘cuvette and electronics holding element’ (Fig. 15b).
3) Screw the LED set up into the ‘cuvette and electronics holding element’ (Fig. 15c).
4) Thread the cable of the LED set up through the hole of the ‘cuvette and electronics holding element’ to the place of the circuit board (Fig. 15d). You might have to enlarge the hole.
5) Fix the circuit board at allocated space with screws (Fig. 15e).
6) Glue the now prepared ‘cuvette and electronics holding element’ onto the ‘smart phone holding element’ with glue (Fig. 15f).
7) Add the 9V-block battery and fix the lid of the electronics part with screws (Fig. 15g).
8) Add the cover of the LEDs and the lid of the cuvette (Fig. 15h). You might have to enlarge the hole for the cable of the earphone plug-in in the cover of the LEDs. HINT: Glue the cover of the LEDs at the end.

This instruction was produced within Citclops. The smart phone add-on was developed and built by the members of the project partner University of Oldenburg, Institute for Chemistry and Biology of the Marine Environment, working group Marine Sensor Systems. Citclops is supported by the EC-FP7 Programme, grant agreement nº 308469. We thank EU for funding!

# SmartFluo >> How to build the smart phone add-on to measure algal fluorescence in water <<

You are done! Now you just combine the add-on with the smart phone; it should look like Figure 16. You hold a powerful fluorescence sensor system in your hands!

**DONE!**

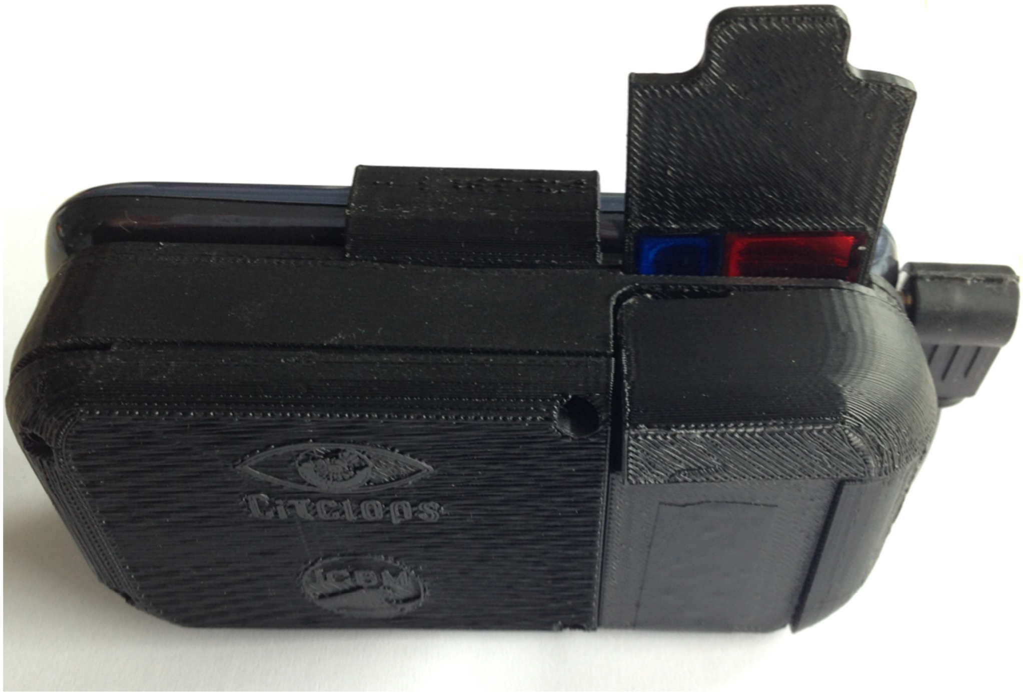


**Fig. 16:** Completed SmartFluo-APP

**To share your data, please prepare a metadata form manually and send form and images to us. Contact person: oliver.zielinski@uni-oldenburg.de**

**For data download use: http://www.citclops.eu/search/welcome.php**

**Thank you for your contribution!**

## Algorithm for interpretation of the fluorescence images

The algorithm (RGB2Chl) for interpretation of fluorescence images is written in Matlab Mathworks® and is available on request.

Please note: Wiley Blackwell is not responsible for the content or functionality of any supporting information supplied by the authors. Any queries (other than missing content) should be directed to the corresponding author for the article

This instruction was produced within Citclops. The smart phone add-on was developed and built by the members of the project partner University of Oldenburg, Institute for Chemistry and Biology of the Marine Environment, working group Marine Sensor Systems. Citclops is supported by the EC-FP7 Programme, grant agreement nº 308469. We thank EU for funding!
